# An audit of Colposcopy referrals from a GU/STD clinic

**DOI:** 10.1186/1756-0500-1-24

**Published:** 2008-06-12

**Authors:** Catherine O'Connor, Helena Myles, Mortimer B O'Connor, Josephine Clancy, Ailis Ryan, Mary Traynor, Dolores McGrath, Kitty O'Sullivan

**Affiliations:** 1 The Dept of GU/STD Medicine, Mid-Western Regional Hospital, Limerick, Ireland; 2 The School of Medicine, University College, Cork, Ireland

## Abstract

**Background:**

Cervical cancer is increasing at 1.5% per year in Ireland with 50% mortality giving 2.2% of all cancer deaths. In the Mid-West region a pilot screening programme has begun to screen all women 25–60 years. 66% of Genitourinary/Sexually transmitted disease (GU/STD) clinics' abnormal smears are <25 years. Requests to abandon "opportunistic" screening prompted this GU/STD clinic audit.

**Methods:**

221(8.4%) patients referred to colposcopy over 4 years were audited. Retrospective analysis was carried out on GU/STD clinic files, hospital files and computer records for biopsy reports. Ethical approval was prospectively granted.

**Results:**

2637 smears were carried out from November 1999 – September 2003.

221 patients referred to colposcopy were audited.

1%, 3%, 5% had severe, moderate and, mild dyskaryosis, respectively, on cervical screening while 0.8%, 1.2%, 1.5% had CIN3, CIN2, CIN1 abnormalities, respectively, on biopsy with 3.5% having no abnormality (Cervical Intraepithelial Neoplasia = CIN).

53% referred to colposcopy were <25 years.

**Conclusion:**

2% had high grade lesions. 37% of high grade lesions are <25 years.

Of the high grade lesions 13% had *Chlamydia trachomatis *(27% of CIN3) and 44% had HPV despite Relative Risks (RR) being 0.75 and 1.09 respectively. Older women had higher grade changes.

No statistical difference was found for progression, regression and persistence in those over and under 25.

## Introduction

Cervical cancer is the second most common cancer in women worldwide and accounts for 273,000 deaths (9% of female cancers) annually and 2.7 million of women life years lost worldwide [1], but only 0.3 million of these are in developed countries. However, the incidence has been greatly reduced in countries where routine Papanicolaou (Pap) testing is accessible. Cervical cancer is increasing at 1.5% per year [2] in Ireland, rising by 36% from 1994–2000, with 50% mortality accounting for 2.2% of all cancer deaths in women [3]. There is lifetime risk of 1 in 25 of developing this cancer, unscreened, by the age of 75. Currently 77 women die in Ireland each year from cervical cancer [4]. Thus it poses an important Public Health problem. The National Cervical Screening Programme (NHSCSP) has reduced mortality in England and Wales [3]. Phase 1 of the Irish Cervical Screening Programme (ICSP) in the Mid-Western Health Board looks promising and is providing a solid foundation for national roll out.

Cytological analysis of cervical smears satisfies most of the Wilson and Junger principles for a screening programme as cervical cancer is prevalent in the community; its natural history is reasonably well understood, with a believed long latency period from HPV infection to invasive disease. Treatment at an early stage is advantageous for the patient and the community, and adequate facilities are available for the diagnosis and treatment of abnormalities identified. However, the Pap cervical smear is not without its problems with sensitivity of ~55% but regular screening may account for the success of the programmes. Problems with this programme have been noted; high risk women have not attended, false negative results (18.5–48%) have been noted. A high grade abnormality does not automatically mean a high grade lesion or visa versa.

A Department of Health Cervical Screening Committee in Ireland reported in 1996 that it is worthwhile to screen 25–60 year olds, 10 years longer than is recommended by World Health Organisation (WHO) and International Agency for Cancer Research (IACR).

The basis for this recommendation is that despite the high rate of abnormal smear results in women under 25 years, invasive cervical cancer is rare in this age bracket [5,6]. Some have argued that over diagnosis, over-treatment and anxiety generated by screening the under 25 age group yields little diagnostic benefits [4]. However, high grade dyskaryosis has increased particularly in <25 year olds (1.3%–1.9%). This accounts for a rise from 15% to 23% of all the high grade lesions [7]. In this GU/STD clinic there is a known 25% abnormality rate in Pap smear (10% ≥ mild dyskaryosis) in the last 18 years where the mean patient age is 23 years. This is similar to a Dublin GU/STD clinic (22%) [8]. It is reported that GUM patients have a higher rate of cytological abnormalities [9]. A request to abandon "opportunistic" screening in the Mid West region outside of the Irish Cervical Screening Programme (ICSP) prompted this audit.

## Methods

All cervical smear results from November 1999 to September 2003 were audited in the GU/STD Clinic, Mid Western Regional Hospital, Limerick. All Pap smears were taken by Ayers spatula and Cytobrush [10,11]. All patients who had a smear with moderate or severe dyskaryosis or had 2 smears with mild dyskaryosis were referred to colposcopy. These patients were identified and their GU/STD and hospital files (hard copy) were audited. Where there was no hospital file or biopsy/lesion report available the histology computer data base was accessed to find biopsy and lesion analysis reports. All demographic details of patients were entered in an Excel spreadsheet, noting age, smear report, referral date, colposcopy impression and biopsy and loop excision reports as well as whether patients had clinically obvious HPV infection or tested positive for *Chlamydia trachomatis(Ct) *(LCx Abbott test). Correlation of Pap smear and biopsies was undertaken.

Relative risks (RR) and 95% confidence intervals (CI) differences were calculated using Excel spreadsheets. Those aged <25 years were compared to the entire group. Histology was taken as the "gold standard". Ethical approval was granted by the Drug and Therapeutic Ethical Committee of the Mid Western Regional Hospital, Limerick, Ireland.

## Results (summarised in table 1)

**Table 1:** Summary of study findings.

	Smear referral	% of all (n = 2637)	Biopsy	% of all (n = 2637)	Biopsy &<25	Biopsy % <25	HPV	<25 yrs	% HPV	Ct	<25 yrs	% Ct
Severe dyskaryosis/CIN3*	29	1%	22	0.8%	7	32%	11	3	50%	6	5	27%
Moderate dyskaryosis/CIN2*	69	3%	32	1.2%	13	41%	13	8	41%	1	0	3%
Mild dyskaryosis/CIN1*	123	5%	39	1.5%	24	62%	29	17	74%	1	1	3%
NAD	0	0	56	3.5%	39	70%	27	23	48%	4	4	7%
not available	0	0	72	2.0%	34	47%	30	24	88%	16	11	22%

Total	221	8.4%	149		117	53%	80	50	54%	17	14	12%

*Dyskaryosis referred to cytology results and CIN to biopsy results.

CIN = Cervical Intraepithelial Neoplasia. NAD = No active disease.

HPV = *Human Papilloma Virus*. Ct = *Chlamydia trachomatis*.

2637 smear results were audited. 7% were deemed "unsatisfactory" for reporting. 221(8%) were referred to Colposcopy. All GU/STD clinic files (221) and 140 hospital files were available for audit. A computer search for reports revealed another 9 reports.

Table 2 shows the varying degrees of dyskaryosis and the histological findings of patients' cervical biopsies and loop excisions taken at colposcopy. Those under 25 years are noted separately.

**Table 2:** Smear and colposcopic findings.

Pap smear results	Smear referral	% total (n = 2637)	<25 years	% total <25 years	% <25 yrs	Biopsy result	No.	<25 yrs	% <25 yrs
Severe Dysplasia	29	1%	9	0.31%	31%	CIN3	10	3	30%
						CIN2	6	1	17%
						CIN1	4	2	50%
						NAD	5	1	20%
						Not available	4	2	50%
Moderate Dysplasia	69	3%	28	1.2%	41%	CIN3	9	3	33%
						CIN2	14	6	43%
						CIN1	16	7	44%
						NAD	16	9	56%
						Not available	14	3	21%
Mild Dysplasia	123	5%	80	66%	66%	CIN3	3	1	33%
						CIN2	12	6	50%
						CIN1	19	15	79%
						NAD	35	29	83%
						Not available	54	29	54%
	221	8.4%	117	4.4%	53%		221	117	

*Dyskaryosis referred to cytology results and CIN to biopsy results.

CIN = Cervical Intraepithelial Neoplasia. NAD = No active disease.

High grade lesions were found in 22%, 42%, 64% of mild, moderate and

severe dyskaryosis while in <25 year olds it was 14%, 36% & 57%.

Biopsy results were accrued and compared for *Human Papilloma*

*Virus *(*HPV*) and *Chlamydia trachomatis *(*CT*).

These results are seen in Table 3.

**Table 3:** Biopsy reports from patients showing all those with *HPV *and *Chlamydia trachomatis *separately. Patients <25 years separated in each group.

Biopsy	No.	HPV+	HPV+ <25 yrs	% HPV+ <25 yrs	HPV+ &Ct+	HPV+ &Ct+ <25 yrs	% HPV+ &Ct+ <25 yrs	Ct+	Ct + <25 yrs	% Ct+ <25 yrs
**CIN3**	22	10	2	20%	1	1	100%	5	4	80%
**CIN2**	32	12	7	58%	1	0	0	0	0	0
**CIN1**	39	29	17	59%	0	0	0	1	1	100%
NAD	56	24	20	83%	3	3	100%	1	1	100%
Not available	72	19	17	90%	11	7	64%	5	4	80%
	149	75	46	61%	5	4	80%	12	10	80%

*Dyskaryosis referred to cytology results and CIN to biopsy results.

CIN = Cervical Intraepithelial Neoplasia. NAD = No active disease.

HPV = *Human Papilloma Virus*. Ct = *Chlamydia trachomatis*.

The RR of patients who attended the clinic with *HPV *and *Ct *were evaluated for referral to colposcopy and having a high grade lesion with these infections.

The results are as follows:

RR of high grade lesion (moderate and severe dyskaryosis) at smear with (1) *HPV *= 1.09 and (2) *Ct *= 0.75. RR of referral to colposcopy from clinic with (3) *HPV *= 1.22, (4) *Ct *= 0.22. However, if referred to colposcopy RR of high grade lesion with (5) HPV = 0.88 and (6) *Ct *= 1.7.

In Table 4 the number of cervical smears that were seen at colposcopy were analysed to see how many progressed, regressed or persisted from Pap smear time.

**Table 4:** Progression, Regression and Persistance seen in each dyskaryotic group. Those under and over 25 were separated.

**Dyskaryosis**	**All Pap Smears**			**<25 years**			**>25 years**			**95% CI of difference**		
	progress	persist	regress	progress	persist	regress	progress	persist	regress	progress	persist	regress

**Severe**		40%	60%		43%	57%		39%	61%		0.62,-0.70	0.6,-0.52
**Moderate**	16%	25%	58%	12%	24%	64%	20%	23%	55%	0.56,-0.40	0.14,-0.16	0.25,-0.43
**Mild**	21%	28%	51%	14%	29%	57%	44%	22%	33%	0.7,-0.121	0.39,-0.53	0.21,-0.69

*Dyskaryosis referred to cytology results and CIN to biopsy results.

CIN = Cervical Intraepithelial Neoplasia.

All smears were analysed together and those over and the under 25 years were separated and 95% CI of the differences between those over and under 25 were calculated.

No difference was seen between the <25 and >25 year olds as both age groups progress, regress and persist at similar rates.

## Discussion

221(8.4%) patients screened were referred to colposcopy of whom 117 (53%) were less than 25 years of age similar to another Irish GU/STD clinic (56%) [8]. 1%, 3%, 5% had severe, moderate and, mild dyskaryosis, respectively, on cervical screening while 0.8%, 1.2%, 1.5% had CIN3, CIN2, CIN1 abnormalities, respectively, on biopsy with 3.5% showing no abnormality.

High grade lesions were found in 25% of mild, 41% of moderate and 61% of severe dyskaryosis. The literature reports that mild, moderate and severe dyskaryosis will give high grade lesions histologically (CIN2 and CIN3) in 50%, 50–75%, 80–90%, respectively, while CIN3 will have 5% invasion rate [11]. Any differences noted may be as a result of the age group of these patients being older or their sample selection. This study population has 37% of all those with high grade lesions (CIN2 +CIN3) <25 years while only 12% was seen in a US study [12]. Older patients (>25 yrs) had a higher prevalence of high grade lesions with 69%, 59%, 34% of CIN3, CIN2, CIN1 being >25 yrs, respectively.

It is accepted Progression, Regression and Persistence have been noted in mild dyskaryosis; 16%, 62%, 22%, moderate dyskaryosis; 35%, 50%,15% [13,14], and regression of 46% [15] in severe dyskaryosis while progression from CIN1 to CIN3 in 26% was seen in a prospective study [16]. In this study the Progression, Regression and Persistence rates for mild dyskaryosis were; 21%, 51%, 28% and moderate dyskaryosis; 16%, 58%, 25% and regression of 60% in severe dyskaryosis. The lower grade lesions had higher persistence and progression while the higher grade lesions had a marginally lower rate of progression and persistence. This was not statistically significant.

*Human Papilloma Virus *(Type 16) has long been associated with cervical cancer. There is a 12% risk of recurrent abnormalities in women with low-risk HPV (non 16 or 18 subtypes) and up to a 50% risk of recurrence in women with certain types of HPV infection (especially subtypes 16 and 18). A change in the disease process in recent years is suggested in findings in the UK and Switzerland [17] where high risk HPV was seen in younger women and may lead to an increase in cervical cancer. In this study, where 28% of women have HPV infection and it is not feasible to type the HPV it is reasonable to postulate that at least 3% of all women seen are probably infected with high grade HPV as it is known that 11% of those with HPV have high grade HPV in China [18] and 7.2% in teenagers in the US where 15% had warts and 11% had *Chlamydia trachomatis *[19].

Transient HPV infection has been postulated for regression rates found. HPV associated changes have been reported in literature in 80% of women ≤ 25 years of age, 66% in the age group 26 to 35 years, 51% in the age group 36 to 45 years and 38% in women aged ≥ 46 years (p = 0.03). In this study there has been a high but not as high a rate of high grade lesions in the >25 year olds (35/54 = 65%) but the rates of progression, regression and persistence are the same in the <25 year olds as was reported by Wright et al in their study of teenagers with 18% having high grade lesions [20].

In this study RR of a high grade lesion with clinical HPV infection was 1.09 but 50% of high grade lesions had HPV. This RR is in agreement with the advice given to women on smear programmes that they do not need to come more often than the general population.

Other Sexually Transmitted Infections have been implicated in causing dyskaryosis especially *Chlamydia trachomatis *[21]. There is 11% prevalence of *Chlamydia trachomatis *in this clinic similar to 9.6% in a Dublin clinic [8]. 27% of biopsy proven CIN3 had Ct in this study and 80% of those were <25 years. However, the RR of having a high grade lesion at time of smear taking with Ct was 0.75 even though 13% of the study's patients with severe dyskaryosis had Ct.

Early sexual debut has not been audited in many studies [22,23]. Younger onset of sexual activity [24] is established in Ireland and may contribute to the earlier age of onset of this cancer. In the 20 patients in this study with high grade lesions sexual debut was available in 8. 5 patients had first sexual intercourse at 14 years or less, one admitted child sexual abuse. Another was 15 and the other 2 were 18 and 19 years of age. These women had a mean number of 5.5 (range 1–20) sexual partners in their lives.

Recent analyses of cost-effectiveness suggest that the addition of molecular HPV DNA testing for women aged over 30 years may allow the screening interval to be lengthened to 3 years for most women but women at high risk for HPV infection and its associated cellular atypias warrant closer monitoring and follow-up. These patients would include organ transplant recipients, women exposed to diethylstilbestrol (DES) and HIV-infected women [25].37% of our patients with high grade lesions were <25 years and 43% of these had HPV and 13% Ct. Should patients at STI clinics be deemed at risk, too, as it was shown in the UK that they have a 10 fold increase in CIN3 (2.3%) [26] in 20–24 year olds compared to the general cervical smear population (0.24%) [27]?

## Conclusion

Cytology-based screening programs for cervical cancer have been effective in reducing cancer incidence and preventing premature deaths worldwide. Although the causal association between infection with certain high-risk types of HPV and the development of cervical cancer has been clearly established, testing for the major risk factor is not part of current screening practice. Screening under 25 year olds has had much discussion.

2% (n = 54) of all patient screened (n = 2,637) had high grade lesions in this study of which 37% (n = 20) were <25 years. 50% of these had HPV while 13% had *Chlamydia trachomatis*. Of the 12 patients with *Chlamydia trachomatis *6 had CIN3. 66% (4/6) were under 25 years. 62% (5/8) of those under 25 with high grade lesions had sexual debut under 14 years of age.

Whether or not HPV, *Chlamydia trachomatis*, early sexual debut or number of sexual partners pre-empts high grade lesions leading to cancer of the cervix needs further evaluation. As GUM patients have a 10 fold increase in CIN3 [26] (20–24 year olds) than the general population in the UK a longer follow up period of these patients would help establish whether STI clinic patients should be included with organ transplant recipients, women exposed to diethylstilbestrol (DES) and HIV-infected women who are advised to have regular re-screening at younger ages.

## Competing interests

The authors declare that they have no competing interests.

## Authors' contributions

COC, HM, MOC carried out data collection, analysed data and prepared paper.

JC AR, MT, DMcG, KOS carried out data collection.

All authors have read and approved the final version of the manuscript
